# Recovery and removal of nutrients from swine wastewater by using a novel integrated reactor for struvite decomposition and recycling

**DOI:** 10.1038/srep10183

**Published:** 2015-05-11

**Authors:** Haiming Huang, Dean Xiao, Jiahui Liu, Li Hou, Li Ding

**Affiliations:** 1 Hebei Key Laboratory of Applied Chemistry, School of Environmental and Chemical Engineering, Yanshan University, Qinhuangdao 066004, PR China; 2 College of Resource and Environmental Engineering, Guizhou Institute of Technology, Guiyang 550003, PR China

## Abstract

In the present study, struvite decomposition was performed by air stripping for ammonia release and a novel integrated reactor was designed for the simultaneous removal and recovery of total ammonia-nitrogen (TAN) and total orthophosphate (P_T_) from swine wastewater by internal struvite recycling. Decomposition of struvite by air stripping was found to be feasible. Without supplementation with additional magnesium and phosphate sources, the removal ratio of TAN from synthetic wastewater was maintained at >80% by recycling of the struvite decomposition product formed under optimal conditions, six times. Continuous operation of the integrated reactor indicated that approximately 91% TAN and 97% P_T_ in the swine wastewater could be removed and recovered by the proposed recycling process with the supplementation of bittern. Economic evaluation of the proposed system showed that struvite precipitation cost can be saved by approximately 54% by adopting the proposed recycling process in comparison with no recycling method.

Nitrogen (N) and phosphorus (P) are the essential elements for the synthesis of nucleic acids and proteins, which are the two most important polymers of life^1^. However, the presence of these elements in substantial quantities in the receiving water body triggers eutrophication, which results in accelerated depletion of dissolved oxygen in the water body and thereby increasing toxicity to the aquatic life. N and P are mainly derived in the water environment from the industrial discharge and municipal wastewaters. Among these wastewaters, swine wastewater simultaneously contains high concentrations of total ammonia nitrogen (TAN) and total orthophosphate (P_T_). Disposal of such inadequately treated wastewater may cause serious environment issues. Therefore, feasible and effective technologies for the treatment of swine wastewater are required for controlling nutrient pollution. In addition, phosphorus is a limited and non-renewable resource. It has been estimated that the available accessible reserves of clean phosphate rock may get depleted within the next 50 years^2^. Currently, there is a growing interest in the technique of removal and recovery of TAN and P_T_ through struvite precipitation by the following reaction^3^,^4^,^5^,^6^.
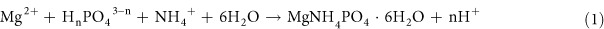


Struvite (MgNH_4_PO_4_ ∙ 6H_2_O), a white crystalline compound [the solubility product (K_sp_) = 7.08 × 10^–14^]^7^, is composed of magnesium, ammonium, and phosphate in equal molar concentrations^8^. This product can naturally crystallize at a pH of 7–11 when the products of ionic activity Mg^2+^, 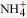, and PO_4_^3–^ exceed the thermodynamic solubility of the struvite product^9^. Struvite precipitation is a technically proven promising technique for the simultaneous removal and recovery of TAN and P_T_. However, economically, TAN removal in the form of struvite does not seem feasible owing to the following reasons^10^: (1) large amounts of external magnesium, phosphate, and alkali are needed to be added to the wastewater; (2) high purity of struvite is difficult to obtain; and (3) the market for struvite as an agricultural fertilizer or industrial materials has not been established.

Disregarding the costs of energy consumption and the associated manpower, the expenditure involved in struvite precipitation for TAN removal includes three costs of magnesium and phosphate sources and that of sodium hydroxide (NaOH). Considering that pure reagents (such as MgCl_2_·6H_2_O and Na_2_HPO_4_·12H_2_O, Mg:N:P = 1:1:1) are used in the reaction, the individual costs of magnesium, phosphate, and NaOH accounted for 10, 81.4, and 8.6%, respectively, of the total cost. Using low-cost magnesium source is thus a crucial approach for cost cutting of the struvite precipitation process^4^,^11^,^12^,^13^,^14^,^15^. Furthermore, since the market price of phosphate salts is usually several times greater than that of the magnesium salts, struvite precipitation cost can be further reduced significantly by using a low-cost phosphate source. However, low-cost phosphate resources are scarce. Moreover, waste liquid containing phosphate is also in much demand^16^. Consequently, it is difficult to reduce this cost to an acceptable level of practical application when the total dependency is placed on the use of low-cost magnesium and phosphate sources for struvite precipitation.

Combining low-cost magnesium and phosphate sources with struvite recycling can reduce the cost of the entire process. The approaches used for struvite recycling mainly include direct pyrolysis^17^,^18^,^19^ and NaOH pyrogenation^8^,^20^,^21^,^22^,^23^. Struvite pyrogenation using both the processes was commonly performed under solid conditions at a temperature of >100 °C. The active products of direct pyrolysis and NaOH pyrogenation of struvite are magnesium hydrogen phosphate (MgHPO_4_) and magnesium sodium phosphate (MgNaPO_4_), respectively, both of which can react with 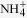 to form struvite. However, irrespective of whether direct pyrolysis or NaOH pyrogenation is performed for struvite decomposition, magnesium pyrophosphate (Mg_2_P_2_O_7_), which exerts egligible effect on TAN removal, is favorably formed due to further dehydration of MgHPO_4_ at temperatures >80 °C^19^,^20^. Moreover, the quantity of Mg_2_P_2_O_7_ in the pyrolysate increases with an increase in the number of recycling, resulting in a rapid reduction in the TAN removal rate^19^,^22^. In the current struvite recycling processes, the transfer and separation of solids between different reactors greatly increases the difficulty of the operation, which results in an increase in the overall treatment cost. To resolve these issues, we proposed a novel struvite decomposition process, which is based on the air stripping technique. This process can lower the decomposition temperature of struvite and stop the formation of Mg_2_P_2_O_7_. Meanwhile, we designed an integrated reactor for the decomposition and recycling of struvite that can save the cost of an individual pyrogenation reactor and avoid the transfer and separation of struvite solids and decomposition products.

The main objectives of the study were to investigate the efficiency and mechanisms of struvite decomposition by air stripping, the performance of TAN removal by recycling the decomposition product, and the removal and recovery efficiencies of TAN and P_T_ via continuous operation of the integrated reactor. For the purpose of reducing the chemical cost of struvite precipitation, i) struvite was obtained from swine wastewater with bittern as magnesium source, ii) the conditions of struvite decomposition by air stripping and the TAN removal from synthetic wastewater by internally recycling struvite decomposition product was studied, iii) a continuous treatment of swine wastewater by using the proposed process with the supplementation of external magnesium source and the recovery of formed struvite was performed, and iv) an economic study of the proposed process was conducted.

## Results and discussion

### Formation of struvite using bittern as a magnesium source

**Figure 1:**
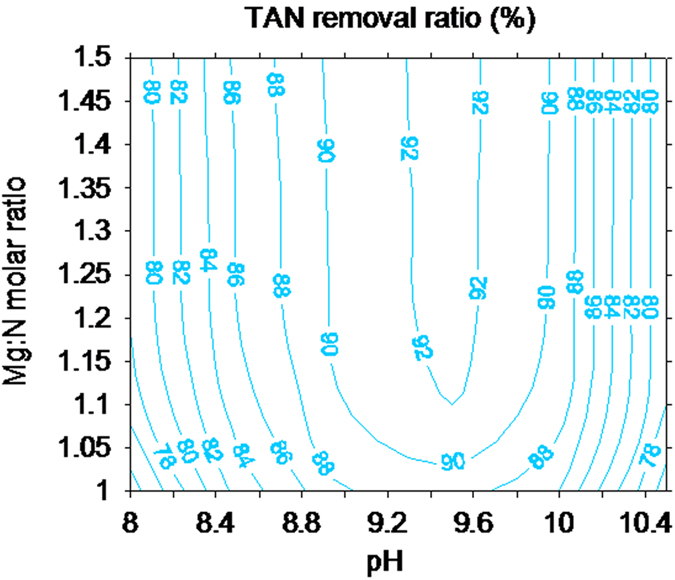
The TAN removal ratio from swine wastewater by struvite precipitation with bittern as a magnesium source at different Mg:N molar ratios (1–1.5) and pHs (8–10.5).

Figure 1 depicts the change in the removal efficiency of TAN with pH values and Mg:N molar ratio. An obvious increase in the TAN removal was noted when the Mg:N molar ratio increased in the range of 1–1.15 at the tested pHs. Nevertheless, a Mg:N molar ratio >1.15 did not incur a further increase in TAN removal. This may be due to insufficient phosphate remaining in the solution for the formation of struvite under the given condition. This finding was in agreement with those in the previous investigations^24^,^25^. Therefore, in this study, proper increase in the dosage of Mg salts was found to be beneficial for TAN removal from swine wastewater.

Solution pH is a key factor that influences the formation of struvite^21^,^26^. It could significantly affect the species of the constitutional ions (Mg^2+^, 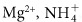, and 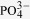) of struvite in the solution and proceeded to control the ionic activity products of Mg^2+^, 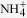, and 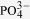. When the practical ionic activity product exceeds the thermodynamic solubility of the struvite product^27^, crystallization of struvite occurs. As shown in Fig. 1, the maximum TAN removal from the swine wastewater was noted at pH 9.5. When the pH increased from 8 to 9.5, the H^+^ concentration rapidly decreased and the proportion of HPO_4_^2–^ species in the solution sharply increased to reach 87% (at pH 8) and 99% (at pH 9) out of the total orthophosphate species^28^. Consequently, this event led to a rapid increase in TAN removal [Eq. (1)]. However, at pH > 9.5, the hydroxide (OH^–^) concentration in the solution increased, and the conversion of 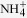 to NH_3_, which cannot be precipitated by the formation of struvite, became even more evident. In addition, at this pH range, some Mg^2+^ in the solution could react with 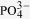 and OH^–^ to form amorphous Mg_3_(PO_4_)_2_ and Mg(OH)_2_^29^, respectively. Moreover, during this reaction, the amount of Mg^2+^ ions involved in struvite crystallization decreased, resulting in a reduction in the TAN removal efficiency.

Different optimum pHs have been reported (8.5^30^, 9^31^, 8.9–9.25^32^, 9.5–10.5^27^) for TAN removal from swine wastewater. In this study, we found that TAN removal from swine wastewater under the conditions of Mg:N:P ratio of 1.15:1:1 and a pH of 9.5 was optimal. Under these optimal conditions, the purity of struvite was estimated to be 93.5%. The levels of magnesium, nitrogen, and phosphorous of the weighed struvite samples were analyzed after the samples were dissolved in 0.5% nitric acid solution, and the Mg:N:P molar ratio was calculated to be 1.09:1:1.06. Thus, the collected struvite was used in the subsequent decomposition experiments.

### Decomposition of struvite by air bubbling

**Figure 2:**
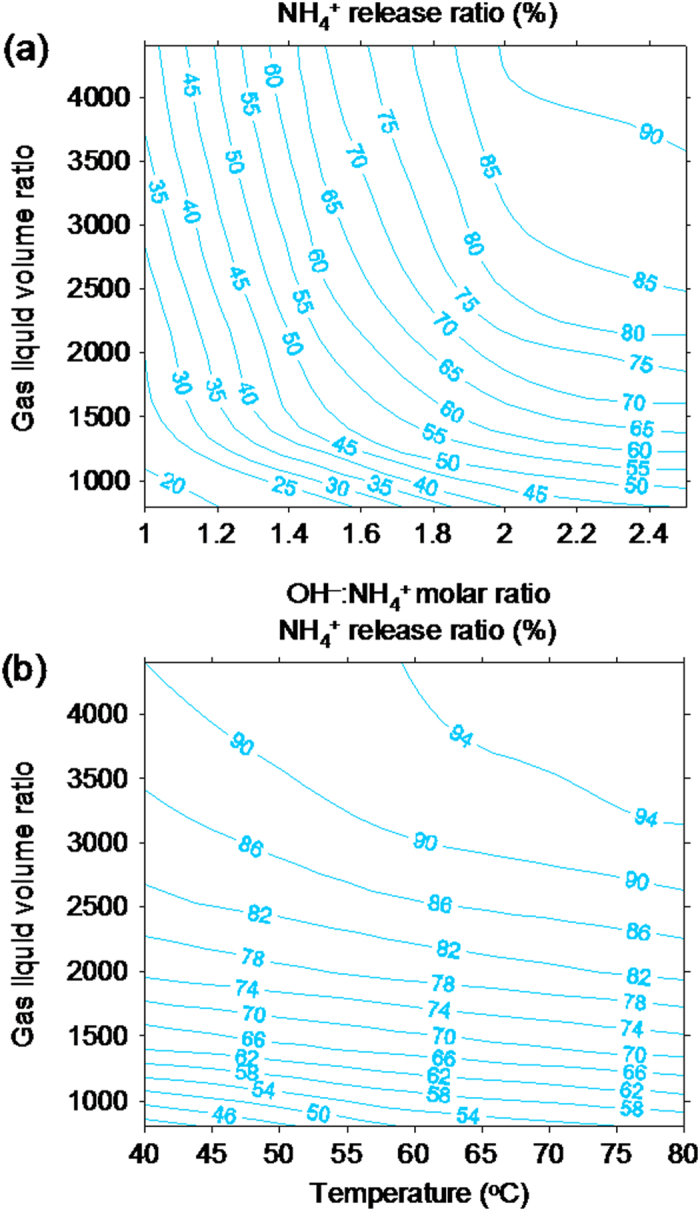
The release ratio of 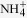 in struvite at different decomposition conditions. (**a**) Effect of 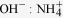 molar ratio (1:1–2.5:1) and gas:liquid ratio (800–4400) on the 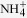 release ratio (40 °C), (**b**) Effect of temperature (40–80 °C) and gas:liquid ratio (800–4400) on the 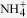 release ratio (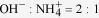).

The effects of gas-liquid (GL) ratio, temperature, and the 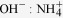 molar ratios on ammonium release were investigated. Figure 2a depicts the changes in the release ratio of ammonium from the solid–liquid system with the 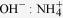 molar ratio and GL ratio at 40 °C. As depicted in Fig. 2a, an increasing 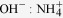 molar ratio and GL ratio resulted in a rapid increase in the ammonium release ratio. When the 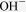 to ammonium molar ratio increased from 1:1 to 2:1 at the tested GL ratios, the ammonium release ratio increased gradually. However, a further increase in the 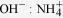 molar ratio ranged from 2:1 to 2.5:1, causing no increase in the ammonium release ratio. At the 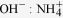 molar ratio of 2:1 and a GL ratio of 3600, the ammonium release ratio was >85%. Figure 2b shows the changes in the ammonium release ratio with the temperature and GL ratios at the 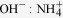 molar ratio of 2:1. Increases in the temperature were accompanied by an increase in the ammonium release ratio. For example, when the temperature increased from 40 °C to 80 °C, the ammonium release ratio increased from 40% to 55% at the GL ratio of 800 and from 87% to 96% at the GL ratio of 3600. Nonetheless, the effect of temperature on the release of ammonium seemed weaker than that by the GL ratio and the 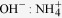 molar ratio. Appropriate increases in the temperature and GL ratios were necessary for effectively enhancing the release of ammonium, although an excess increase in these parameters may not be economical for a small increase in ammonium release. In this study, we found that controlling the GL ratio at 3600, temperature at 60 °C, and the 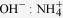 molar ratio at 2:1 was economical and reasonable. Under these conditions, the ammonium release ratio reached >92%.

Considering struvite decomposition, several methods including NaOH pyrogenation (decomposing struvite at the solid–solid system)^21^,^22^, direct pyrolysis^17^,^18^,^22^, acidolysis^33^, chlorination decomposition^34^, and electrolytic decomposition^35^ were reported. As for NaOH pyrogenation of struvite, He *et al.*^8^ reported that >96% of ammonium could be released from struvite under the 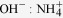 ratio of 1:1, temperature of 90 °C, and a heating time of 2 h. Tüker and Çelen^20^ found that pyrolyzing struvite at the 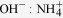 ratio of 1:1 and at 110 °C for 3 h gave an ammonium release ratio of 81%. Zhang *et al.*^21^ considered that, when struvite was decomposed at an 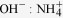 molar ratio of 2:1 at 110 °C for 3 h, an ammonium release ratio of >92% was achieved. Yu *et al.*^23^ also reported that the best pyrogenation condition using a 1:1 ratio of NaOH powder:ammonium at 110 °C for 3 h; under these conditions, the ammonium release ratio reached >96%. Our findings were comparable to that of NaOH pyrogenation in previously reported studies. In addition, the ammonium release ratio by the proposed decomposition method of struvite approached those by other decomposition methods, for example, that of 98% ammonium release ratio by acid dipping^33^ and chlorination decomposition methods^34^.

### Product characterization and decomposition mechanism

**Figure 3:**
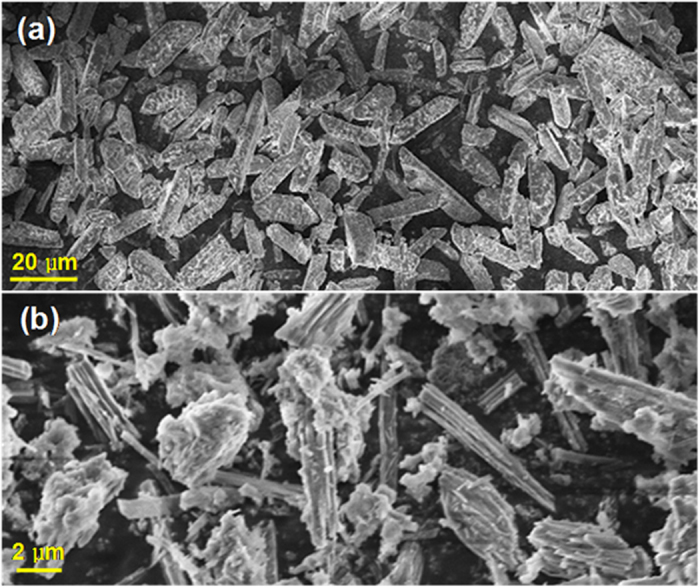
Scanning electron microscopy of the solid components before (**a**) and after (**b**) struvite decomposition.

**Figure 4:**
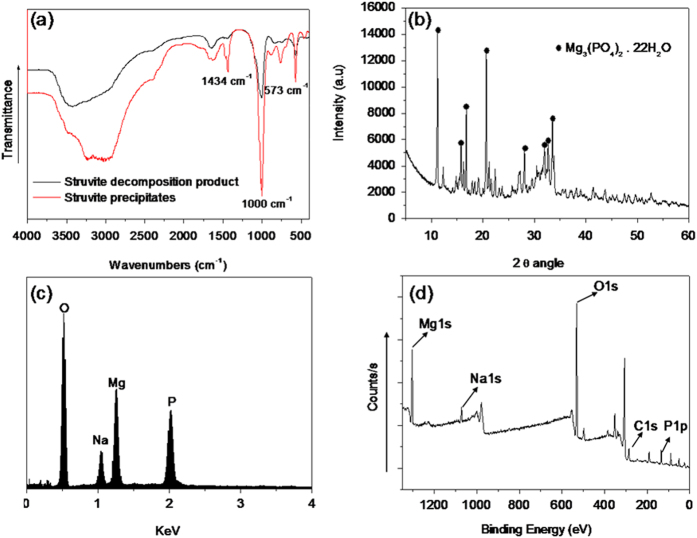
(**a**) FTIR spectroscopy of the solids before and after struvite decomposition, (**b**) XRD pattern, (**c**) EDS spectrogram, (**d**) and XPS pattern of the solid obtained at the end of struvite decomposition.

The solid product obtained at the optimal decomposition conditions was first characterized by SEM, XRD, and FTIR. SEM analysis (Fig. 3) revealed that the solid morphology before and after struvite decomposition changed from the original rectangular block shape to the final needle shape, suggesting that some crystalline products may have been formed. FTIR analysis results (Fig. 4a) revealed that the scissoring vibration of 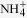 at approximately 1435 cm^−1^ disappeared in the spectroscopy of the solid product, while the scissoring vibration of PO_4_ remained at approximately 573 cm^−1^ and 1004 cm^−1^; this result implied the release of ammonium from struvite. Nevertheless, in the FTIR spectroscopy, the characteristic bands of P–O–P (Mg_2_P_2_O_7_) at approximately 889 cm^−1^ was not detected, which indicated that Mg_2_P_2_O_7_ was not a derivative of struvite decomposition by the proposed method. Mg_2_P_2_O_7_ was favorably formed in struvite pyrogenation at >80 °C and under solid conditions^8^,^20^,^23^. However, in this study, the decomposition conditions did not support the formation of Mg_2_P_2_O_7_. In addition, XRD characterization (Fig. 4b) revealed that the main composition of the decomposition product was magnesium phosphate 22-hydrate [Mg_3_(PO_4_)_2_·22H_2_O]. To further determine the composition of the decomposition product, EDS and XPS analyses of the solid product were conducted and the supernatant composition at the end of the reaction was determined. The EDS and XPS spectrums (Fig. 4c,d) revealed the peaks of Mg, Na, P, and O, suggesting that MgNaPO_4_ may be a derivative of struvite decomposition. In addition, the results of the chemical analysis revealed that the pH and concentrations of Mg and P_T_ of the supernatant at the end of the reaction were 11.2 ± 0.05, 23.5 ± 2.1 mg/L, and 1408 ± 69 mg/L, respectively. On the basis of the mass balance before and after struvite decomposition, the Mg:P molar ratio of the remaining solid after decomposition can be calculated to be approximately 1.4, which is near 1.5 of Mg_3_(PO_4_)_2_. This result is consistent with the results of XPS analysis. Therefore, based on the results of characterization and chemical analysis, MgNaPO_4_ was determined to be one of the solid products formed in the decomposition process. Moreover, in the solution with a pH value > 11, Mg(OH)_2_ may also be one of the derivatives.

The structure of struvite is orthorhombic, that is, it is composed of PO_4_^3–^ tetrahedral, Mg(6H_2_O)^2+^ octahedral, and 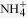 groups held together by hydrogen bonds^36^. When NaOH was added to the struvite mixture, a 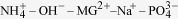 solid–liquid system was formed. In this system, the 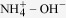 (i.e., NH_3_·H_2_O) structure was unstable. NH_3_ was readily released on the GL mass transfer in the solution. Increases in the temperature and GL ratio was accompanied by the rapid release of NH_3_. Therefore, under this high alkaline condition, Mg_3_(PO_4_)_2_·22H_2_O could be favorably formed along with small amounts of Mg(OH)_2_. In addition, in the solid–liquid system, Na^+^ could replace 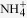 to form MgNaPO_4_·7H_2_O, which is an isomorphous analogue of struvite. Several other analogues such as MgKPO_4_·6H_2_O, MgRbPO_4_·6H_2_O, MgTlPO_4_·6H_2_O, and MgCsPO_4_·6H_2_O have also been reported^37^,^38^. The general formula of these analogues can be described as MgMPO_4_ (*M* representing positive monovalent ions). The stability of MgMPO_4_ was closely related to the ionic radius of M in MgMPO_4_, and it generally increased with an increase in the radius of the univalent ion. Consequently, the 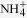, Tl^+^, and Cs^+^ crystals showed higher stability than K^+^, Na^+^, and Rb^+^ crystals^23^,^38^.

The cumulative results suggest that the overall reaction occurring in the solid–liquid system during struvite decomposition by the proposed process can be expressed as follows:
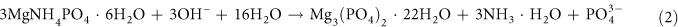

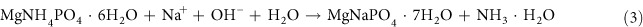

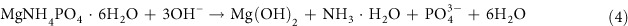

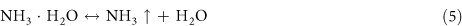


### Performance of recycling of struvite decomposition product

#### Optimal pH of recycling the decomposition product

**Figure 5:**
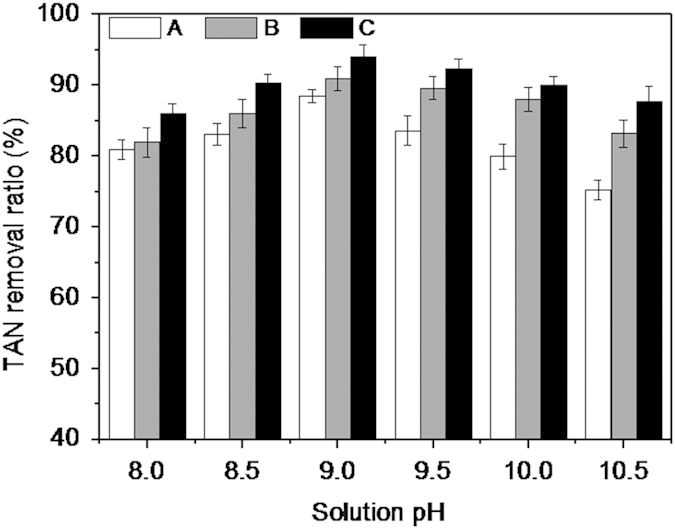
The TAN removal ratio of struvite decomposition product at different pHs. (**a**) TAN removal with direct reuse of the decomposition product. (**b**) TAN removal with the acidification of the decomposition product. (**c**) TAN removal with the acidification of the decomposition product and the addition of seeding material.

To investigate the performance of recycling the struvite decomposition product at different pHs (8–10.5), three modes of reuse of the struvite decomposition product were adopted. In the first mode, the decomposition product was directly reused. In the second mode, the solution pH of the decomposition product was adjusted to 6 before reuse, and then the pretreated solution was reused for TAN removal. In the third mode, the pretreated product of the second mode was reused along with pre-formed struvite as the seeding material (5 g of preformed struvite per liter of wastewater). The experimental results are shown in Fig. 5. The TAN removal ratios of the three modes were found to increase in the pH range of 8.0–9.0, reaching the maximum at pH 9, followed by a decrease in the pH from 9.5 to 10.5. At the identical pH, the TAN removal ratio of the third mode was the highest, followed by the second and the first mode. Struvite crystallization was controlled by two mechanisms: crystal nucleation and crystal growth^39^. The use of crystal seed could shorten the induction time of struvite crystallization (crystal nucleation) and promoted the rapid growth of struvite crystal (crystal growth), resulting in an increase in the rate of ammonium removal^21^. For example, Kim *et al.*^39^ reported that, when the dosage of the preformed struvite was increased from 0 to 40 g/L, the removal ratio of TAN from the landfill leachate increased from 88% to 98%.

In the first mode, the reaction equations for the formation of struvite may involve Eq. (1) and the following equations:
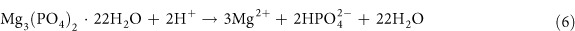

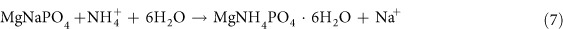

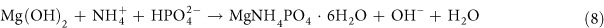


Notably, although Mg_3_(PO_4_)_2_·22H_2_O was the main composition of the solid product, a high removal efficiency of TAN (approximately 89% at pH 9) was noted in the recycling of the decomposition product in the first mode; this finding was inconsistent with those of Sugiyama and Yokoyama^19^, who reported that Mg_3_(PO_4_)_2_ has a weak effect on TAN removal. However, this inconsistency can be attributed to the use of Mg_3_(PO_4_)_2_, an amorphous solid matter, in the previous study and the use of Mg_3_(PO_4_)_2_·22H_2_O, a crystalline matter, in the present study. It has been reported that Mg_3_(PO_4_)_2_·22H_2_O is unstable in water and can be dissolved as depicted in Equation 6 at a low pH range^7^,^40^. As a result, in the recycling process, Mg_3_(PO_4_)_2_·22H_2_O was favorably transformed to Mg^2+^ and HPO_4_^2–^, which could be used for struvite formation. As the stability of MgNaPO_4_ is lesser than that of MgNH_4_PO_4_, when MgNaPO_4_ was added to the aqueous solution containing ammonium, Na^+^ in the MgNaPO_4_ could be replaced by 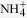 to form MgNH_4_PO_4_·6H_2_O.

In the second and third modes, the Mg(OH)_2_ and phosphate compounds in the decomposition product were converted to MgHPO_4_ and dissolved Mg^2+^ and H_2_PO_4_^–^ when the product solution pH was adjusted to 6. Therefore, in both the modes, the TAN removal process proceeded according to Eq. (1) and Eq. (9), as follows:
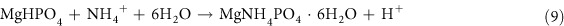


In this study, acidolysis of the decomposition product and the addition of preformed struvite as a seeding material could increase the rate of TAN removal (approximately 6%), which is not feasible from the economical perspective. Hence, the direct reuse of struvite decomposition product was performed in the subsequent experiments.

#### Multiple recycling of decomposition product

**Figure 6:**
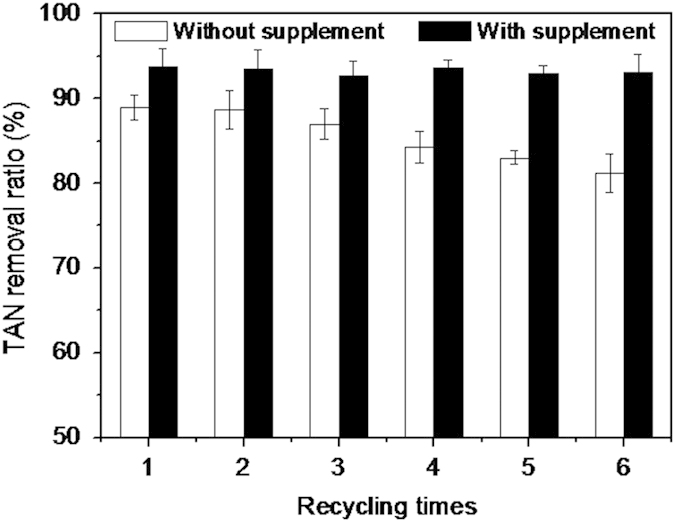
The TAN removal ratio of struvite decomposition product at different recycling times.

During the multiple recycling of struvite decomposition product, two recycling modes were conducted to investigate the performance of the decomposition product as magnesium and phosphate sources for TAN removal from simulated wastewater. First, the decomposition product was used directly and repeatedly at pH 9. Second, the decomposition product was reused with the supplementation of bittern and Na_2_HPO_4_·12H_2_O at the Mg:N:P molar ratio of 0.1:1:0.1 in each cycle (at pH 9). The experimental results are described in Fig. 6. It was observed that, with an increase in the number of times of recycling, the TAN removal ratio in the second mode was constantly maintained at approximately 93%, while that of the first mode decreased progressively. The TAN removal ratio without supplementation of external magnesium and phosphate sources was 89% in the first cycle and 81.2% in the sixth cycle. In the first mode, the decrease in TAN removal rate could have been due to the losses of Mg^2+^ and 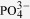 in the supernatant in each recycling cycle. In addition, inactive amorphous Mg_3_(PO_4_)_2_ formed and accumulated during the recycling of struvite decomposition product may also be responsible for this decreased rate^41^. Nevertheless, due to the absence of Mg_2_P_2_O_7_ in the decomposition product, the TAN removal ratio by the proposed process did not decrease rapidly as in other investigations; instead it reduced by only (approximately) 8% after six recycling cycles. Türker and Çelen^20^ reported that the TAN ratio was initially 92%, which progressively decreased to 77% in the fifth recycle cycle. Huang *et al.*^22^ recycled the struvite pyrolysate five times and found that the TAN removal ratio decreased from 80% in the first cycle to 67% in the fifth cycle. He *et al.*^8^ investigated the TAN removal ratio from landfill leachate by recycling the NaOH pyrolysate of struvite and reported that it rapidly decreased from >90% in the first cycle to <65% in the sixth cycle. Therefore, in terms of multiple recycling of the struvite decomposition product, the proposed process demonstrated a preferable performance on TAN removal in comparison with the other struvite recycling methods^8^,^21^,^22^,^23^.

### Continuous treatment of swine wastewater by the integrated reactor

**Figure 7:**
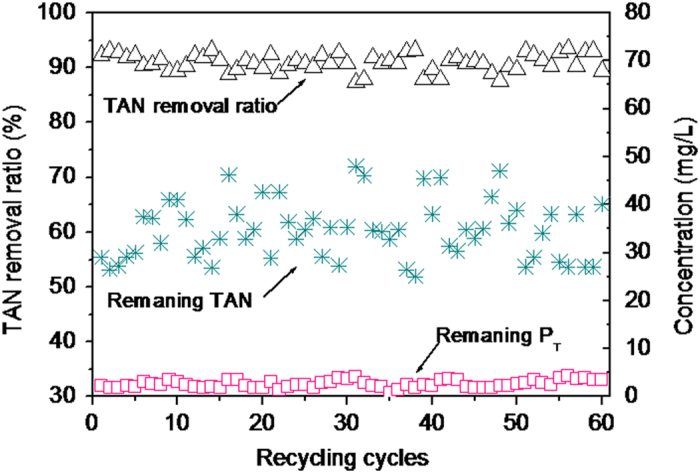
The continuous treatment of swine wastewater by the proposed struvite recycling process in the integrated reactor.

The continuous removal and recovery of TAN and P_T_ from real swine wastewater were conducted under the optimal conditions. The results of continuous operation for 60 cycles are shown in Fig. 7. We found that the TAN concentration of the effluent was maintained at approximately 34 mg/L, with an average removal efficiency of approximately 91 ± 3.5% standard deviation. In addition, the remaining P_T_ concentration remained stable at approximately 3 mg/L. The recovery efficiency of P_T_ can be calculated to be approximately 97%. In this continuous operation, through the supplementation of an external bittern source, the P_T_ originally present in the swine wastewater could be fully utilized as the phosphate source for TAN removal in the recycling process of struvite decomposition product. Similar result was obtained in the process in which magnesium and phosphate were externally added at a Mg:N:P molar ratio of 0.12:1:0.12 in each recycling cycle. Our results, detailed in the multiple recycling of decomposition product, suggested that the supplementation of external magnesium and phosphate source could markedly improve TAN removal. However, this proposed continuous process did not consume additional phosphate sources and increase the operation difficulty and treatment costs. In addition, the precipitates collected during the continuous operation were characterized by SEM and XRD to reveal needle-shaped crystals, mainly composed of struvite. The chemical composition of the precipitates was also analyzed after dissolution in 0.5% nitric acid solution, indicating that the purity of struvite precipitates was 92.5% (±0.6) and the contents of Mg, N, P, K, and Ca were 98.8 ± 1.1, 52.9 ± 0.5, 124.3 ± 0.6, 2.2 ± 0.8, and 4.3 ± 1.1 mg/g, respectively.

Previous studies designed reactors dealing with the removal and recovery of TAN and P_T_ from swine wastewater^42^,^43^,^44^,^45^,^46^. Shepherd *et al.*^46^ reported a full-scale, continuous-flow struvite precipitation reactor with air sparging for pH adjustment and mixing, which could provide 95% reduction in the phosphate concentration from swine wastewater. Suzuki *et al.*^42^ developed a continuous-flow reactor with dual functions of achieving crystallization of struvite through CO_2_ stripping and separating the formed struvite; they could recover 72.8% of phosphate from swine wastewater by using the designed reactor. Song *et al.*^44^ designed a continuous-flow reactor equipped with struvite accumulation devices and found that, up to 85% phosphate was immobilized as struvite and 40–90% TAN was removed via air stripping by a reactor without addition of any chemicals. Huang *et al.*^45^ introduced a continuous-flow reactor with a removal capacity of approximately 93% TAN from swine wastewater by using a seeding crystal technique. In this study, although our designed integrated reactor mainly focused on TAN removal by struvite recycling, we simultaneously achieved a recovery efficiency of phosphate comparable to those reported earlier. From the experimental results, we can conclude that the proposed process was technically feasible.

### Economic evaluation

To further determine the economical feasibility of the proposed recycling process, the cost of removing 1 kg TAN from swine wastewater was evaluated and compared to that of struvite precipitation using bittern and pure Na_2_HPO_4_·12H_2_O (Mg:N:P = 1.15:1:1) without struvite recycling. This economic evaluation excluded the related costs due to manpower and struvite and only included the costs of chemicals and energy consumed in the process. The market prices of the chemicals and energy consumed and the cost distributions of each of the constituents are given in Table 1. The bittern used in this study was the byproduct of salt manufacturing process, thus the actual cost incurred was related to transportation, which was very low for the bittern of unit volume. In addition, the amount of bittern consumed in the treatment was extremely small. Hence, the cost of bittern in the calculation was also not considered. The calculation results listed in Table 1 indicate that the cost for removing 1 kg TAN from swine wastewater by using the proposed recycling process was 4.54 USD, whereas that for struvite precipitation without recycling of struvite was 9.83 USD. Compared to that in struvite precipitation process without struvite recycling, approximately 54% of cost can be saved by using the proposed recycling process. Hence, the use of the proposed process not only enabled cost savings but also achieved the recovery of phosphate from swine wastewater.

With regard to the recycling technique of struvite, He *et al.*^8^ used NaOH pyrogenation technique for struvite recycling and achieved 44% cost saving (for chemicals) by recycling the pyrolysate of struvite for three cycles. Liu *et al.*^35^ reported that approximately 60% of the chemical costs were reduced by recycling the electrochemical decomposition product of struvite. Huang *et al.*^16^ introduced a chlorination decomposition technique of struvite and saved approximately 34% of the cost by it. Compared with other studies, it was found that cost saving by the proposed struvite recycling process in this study was evidently higher than that by NaOH pyrogenation of struvite and that it was close to that of the electrochemical decomposition of struvite. In addition, although the cost of the proposed recycling process was higher than that (3.06 USD/kg TAN) of the electrochemically regenerated ion exchange process^47^, the struvite recycling process with the use of the designed integrated reactor had some additional advantages such as simplicity of operation and the low cost of equipment. Thus, the proposed process was economically feasible.

## Materials and experimental methods

### Swine wastewater and bittern properties

Swine wastewater was collected from a pig farm located in the suburb of Beijing. The collected wastewater was filtered through a filter paper to remove the suspended solids and then stored in a refrigerator at 4 ± 1 °C. Bittern (used as the magnesium source) was collected directly from a solar salt field in Tianjin; its chemical composition was identical to that reported by Huang *et al.*^34^. Table 2 presents the average properties of swine wastewater used in this study.

### Design of integrated reactor

**Figure 8:**
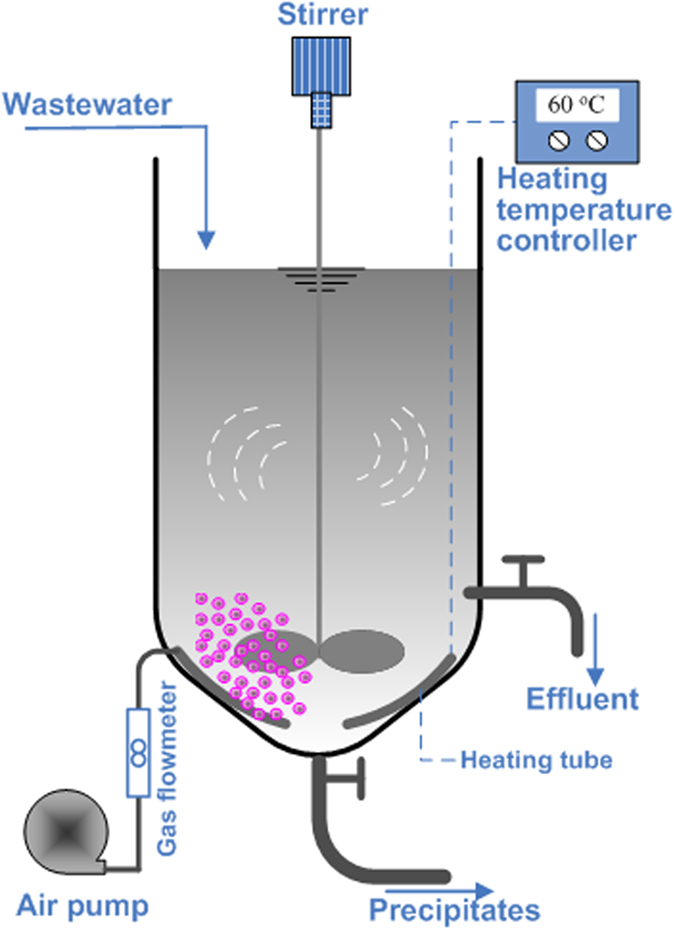
Schematic drawing of the integrated reactor for precipitation and struvite recycling.

To achieve the simultaneous removal and recovery of TAN and P_T_ from swine wastewater by internally recycling struvite decomposition product, an integrated reactor was designed, which can be simultaneously used for the treatment of wastewater and struvite decomposition. The schematic diagram of the reactor is shown in Fig. 8. The reactor was a 1.2-L barrel with a conical bottom consisting of a 0.85-L reacting zone and a 0.15-L settling (or decomposing) zone (Fig. 8). Stirring was performed using a stirrer fixed in the reactor. At the end of the struvite precipitation reaction, the formed precipitates could be settled at the settling zone (150 mL), while the supernatant (850 mL) was discharged from the outlet set at the side of the reactor. Subsequently, the solid and the solution accumulated in the settling zone were used to perform struvite decomposition. To assure ready escape of ammonia, an air bubble diffuser with micro-orifices (*Φ* = 0.2 mm) present in the tube was mounted at the bottom of the reactor to provide diffused aeration for ammonia stripping. Meanwhile, a heating tube with a temperature controller was set at the bottom of the reactor to control the solution temperature at the desired range. In addition, another outlet was installed at the bottom of the reactor for precipitate collection.

### Experimental methods and conditions

#### Formation of struvite

Struvite was produced at an ambient laboratory temperature of 15–20 °C. Briefly, 1000 mL of swine wastewater was fed into the integrated reactor. Then, bittern and pure Na_2_HPO_4_·12H_2_O was added to the wastewater at the desired Mg:N molar ratio (at a constant N:P molar ratio of 1:1). Next, the mixture was stirred at a stirring rate of 300 rpm and at a given pH (8, 8.5, 9, 9.5, 10, and 10.5) for 30 min and the solution obtained at the end of reaction was allowed to settle for 60 min. Finally, the supernatant was filtered through a 0.45-μm membrane filter for component analysis.

#### Decomposition of struvite

Under the abovementioned optimal conditions for struvite formation, the solid-liquid mixture accumulated in the settling zone of the reactor was used to decompose struvite via air stripping. Briefly, the temperature of the mixed solution was adjusted as desired (40, 60, and 80 °C), NaOH powder was added to the mixture (approximately 150 mL) at a given 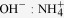 molar ratio (1:1, 1.5:1, 2:1, and 2.5:1), the reaction mixture was stirred at a stirring rate of 200 rpm to assure proper homogenization of the mixture, and an air pump was used to pump in air in the system as well as push into the mixture at a different gas-liquid (GL) volume ratio (800–4400). Finally, the pH of the mixed solution at the end of reaction was adjusted to 3 with 0.5% nitric acid to completely dissolve the solid matter, and 5 mL of the resulting solution was collected for component analysis. Since ammonia recovered from a solution could be easily adsorbed by water or acid solution^48^, its further treatment was not investigated in this study.

#### Recycling of struvite decomposition product

The TAN removal performance of the decomposition product obtained under the optimal conditions was investigated at the experimental pH of 8–10.5. The wastewater used in the experiments was synthetic wastewater with a TAN concentration equal to that of swine wastewater, which was prepared by dissolving NH_4_Cl of analytical grade into pure water. Synthetic wastewater was added to the solution of the decomposition product at the TAN:P_T_ molar ratio of 1:1 (TAN in the wastewater to the P_T_ in the mixture). The experiments were performed as detailed in the section of formation of struvite. For multiple recycling of the struvite decomposition product, (1) struvite crystal was prepared according to the optimal conditions detailed in earlier section, (2) the collected precipitates was decomposed under optimal conditions mentioned in the section of decomposition of struvite, (3) the decomposition product of struvite was recycled for TAN removal from the synthetic wastewater at the optimal pH obtained from the above experiments, and the steps (2) and (3) were repeated for several cycles. The composition of the effluent was analyzed as described earlier.

### Continuous operation of an integrated reactor

Based on the results of the abovementioned experiments, the continuous operation experiments were performed to investigate the stability for the removal and recovery of TAN and phosphate by the proposed struvite recycling process. In this experiment, swine wastewater was used. The operation procedures were identical to those of multiple recycling detailed in the section on recycling of struvite decomposition product. However, in order to maintain stability in the removal and recovery efficiencies of TAN and phosphate from swine wastewater, bittern was supplemented at the Mg:N molar ratio of 0.12:1 at each recycling cycle. Due to the presence of phosphate in the original swine wastewater, addition of external magnesium source led to an increase in the amount of struvite in the reactor. Calcium and potassium present in swine wastewater and inactive derivatives such as amorphous magnesium phosphate formed at struvite crystallization and decomposition process may be progressively accumulated in the struvite precipitates, which can lower the performance of recycling of the struvite decomposition product, a certain amount of struvite formed was discharged from the outlet installed at the bottom of the reactor at each recycling cycle to maintain the amount of active magnesium and phosphate constant and control the amount of calcium and potassium in the precipitates at stable ranges. During this continuous operation, the supernatant formed at the end of each recycling cycle was collected and filtered through a 0.45-μm membrane filter for component analysis.

### Analytical methods

The composition of wastewater samples was determined by the standard methods detailed elsewhere^49^. TAN and P_T_ were measured by the Nessler’s reagent spectrophotometric method and Mo–Sb anti-spectrophotometric method (752 N-spectrophotometer; China), respectively. The concentrations of metal cations such as K^+^, Ca^2+^, Na^+^, and Mg^2+^ were analyzed by using an atomic adsorption photometer (AA-6800; Shimadzu, Japan). In this study, the solution pH was measured by using a pH meter (pHS-3C; China). The solids collected at the end of reaction were washed with deionized water thrice, followed by oven drying at 35 °C for 48 h. The morphology of dried solids was observed under a scanning electron microscope-energy dispersive spectrometer (SEM-EDS; SUPRA 55 SAPPHIRE; Germany). The composition of the solids was analyzed by X-ray diffraction analyzer (XRD; DMAX-RB; Rigaku, Japan), X-ray photoelectron spectrometer (XPS; Phi-5000 Versa Probe; ULVCA-PHI, USA), and fourier-transformed infrared spectroscopy (FTIR; NEXUS870, USA). In this study, the volume change in a solution incurred by air stripping was included in the calculation. All tests were performed in triplicates, and their average data was reported.

## Author Contributions

H.H.M. planned the project and data analysis. X.D.A., D.L., and L.J.H. performed the experimental works and prepared all the figures. H.L. performed the supplementary experiments. All authors discussed the data, interpreted the results, and jointly wrote the paper.

## Additional Information

**How to cite this article**: Huang, H. *et al*. Recovery and removal of nutrients from swine wastewater by using a novel integrated reactor for struvite decomposition and recycling. *Sci. Rep.* **5**, 10183; doi: 10.1038/srep10183 (2015).

## Figures and Tables

**Table 1:** The market price of chemicals and energy consumed and the cost of the proposed recycling process and struvite precipitation without recycling.

**Chemical/energy**	**Market price (US$/kg)**	**Cost of struvite precipitation without recycling (US$/kg TAN)**	**Cost of the proposed recycling process (US$/kg TAN)**
Na_2_HPO_4_·12H_2_O	0.36	8.90	—
NaOH	0.29	0.93	1.74
HCl (31%)	0.07	—	0.47
Energy	0.1 kWh	—	2.33
Total	—	9.83	4.54

**Table 2:** Average properties of swine wastewater used in this study.

**Parameter**	**Value and S.D.**
pH	7.7 ± 0.2
Alkalinity (as CaCO_3_) (mg/L)	1639 ± 126
COD (mg/L)	2756 ± 184
TAN (mg/L, as N)	378 ± 24
P_T_ (mg/L, as P)	105 ± 8.4
K (mg/L)	342 ± 21
Ca (mg/L)	39 ± 9
Mg (mg/L)	21 ± 5
Fe (mg/L)	0.9 ± 0.1
Na (mg/L)	276 ± 31
Cl^–^ (mg/L)	563 ± 48
